# Longitudinal tracking of circulating rare events in the liquid biopsy of stage III–IV non-small cell lung cancer patients

**DOI:** 10.1007/s12672-024-00984-4

**Published:** 2024-05-03

**Authors:** Lily Bai, George Courcoubetis, Jeremy Mason, James B. Hicks, Jorge Nieva, Peter Kuhn, Stephanie N. Shishido

**Affiliations:** 1 Convergent Science Institute in Cancer, Michelson Center for Convergent Bioscience, University of Southern California, Los Angeles, CA 90089 USA; 2 Department of Biological Sciences, Dornsife College of Letters, Arts, and Sciences, University of Southern California, Los Angeles, CA 90089 USA; 3 Catherine and Joseph Aresty Department of Urology, Institute of Urology, Keck School of Medicine, University of Southern California, Los Angeles, CA 90033 USA; 4 Norris Comprehensive Cancer Center, Keck School of Medicine, University of Southern California, Los Angeles, CA 90033 USA

## Abstract

In the United States, lung cancer is the second most common type of cancer with non-small cell lung cancer (NSCLC) encompassing around 85% of total lung cancer cases. Late-stage patients with metastatic disease have worsening prognosis, highlighting the importance of longitudinal disease monitoring. Liquid biopsy (LBx) represents a way for physicians to non-invasively track tumor analytes, such as circulating tumor cells (CTCs), and understand tumor progression in real-time through analyzing longitudinal blood samples. CTCs have been shown to be effective predictive biomarkers in measuring treatment efficacy and survival outcomes. We used the third-generation High-Definition Single Cell Assay (HDSCA3.0) workflow to analyze circulating rare events longitudinally during treatment in a cohort of 10 late-stage NSCLC patients, identifying rare events including circulating cancer cells (i.e., CTCs), and oncosomes. Here, we show (1) that there is a cancer specific LBx profile, (2) there is considerable heterogeneity of rare cells and oncosomes, and (3) that LBx data elements correlated with patient survival outcomes. Additional studies are warranted to understand the biological significance of the rare events detected, and the clinical potential of the LBx to monitor and predict response to treatment in NSCLC patient care.

**Supplementary Information:**

The online version contains supplementary material available at 10.1007/s12672-024-00984-4.

## Introduction

Non-small cell lung cancer (NSCLC) is a subtype of lung cancer and accounts for 80–85% of total lung cancer cases [1]. In the United States, lung cancer is the second most common cancer type [1, 2]. The overall 5-year survival rate of NSCLC is around 23% for men and 33% for women, but greatly declines with disease progression [3]. Late-stage patients with metastatic disease have worsening prognosis, highlighting the importance for longitudinal monitoring to better understand progression and assist in clinical decision making. Current clinical standards for monitoring NSCLC include imaging (MRI scans, CT scans, and PET scans) and lung biopsies or thoracentesis, procedures that are invasive and may add risk and anxiety for the patient. Therefore, there exists a need to monitor patients in a minimally invasive, longitudinal manner, which may allow for real-time information on disease progression and response to treatment.

Liquid biopsy (LBx) has emerged as a way to enhance understanding of disease progression in NSCLC [4–8]. LBx allows for collection of longitudinal samples in a minimally invasive route for analysis of multiple analytes. Within NSCLC, cell-free DNA (cfDNA) has commonly been used to understand disease progression and patient prognosis [9–12]. CfDNA is fragments of DNA found in the blood and has been a source of genomic information on the tumor itself. In a prospective observational study of over 300 individuals at risk of developing lung cancer, 129 were determined to have lung cancer using cfDNA fragments showing that cfDNA fragments from NSCLC patients displayed widespread genome wide variations and are associated with decreased overall survival (OS) [13, 14]. CfDNA is primarily used given the rarity of circulating tumor cells (CTCs) in NSCLC.

Despite its rarity, CTCs remain a useful prognostic tool and have been indicated as a predictive biomarker associated with progression free survival (PFS) and OS for NSCLC patients [4, 7, 8, 15–18]. Currently, the only LBx assay approved by the U.S. Food and Drug Administration (FDA) is CellSearch, which is a platform that uses EpCAM enrichment for the detection of CTCs in patients with colorectal, breast, or prostate cancer [19]. In NSCLC, CellSearch detected CTCs in only around 1/3 of metastatic stage patients at baseline in a study done by Tamminga et al. [20]. While Krebs et al. observed that CellSearch CTC number was the strongest predictor of OS in a cohort of 101 stage III and IV NSCLC patients [21]. Enrichment based approaches are biased in the type of cells detected based on a predetermined definition of a CTC. Other rare cell types, including circulating megakaryocytes and endothelial cells have been linked to survival implications [22–25] and are found in NSCLC [26, 27]. Herein, we present a single-cell, non-enrichment approach to allow for the detection of all rare events found in the blood.

Here, we use the High-Definition Single Cell Assay (HDSCA3.0) for high-throughput analysis of epithelial, mesenchymal, endothelial, and immune cells with detection and characterization of rare events, including CTCs, tumor microenvironment, and oncosomes (or large extracellular vesicles), from the peripheral blood of patients with stage III-IV NSCLC throughout treatment. Using this methodology, we have previously identified and characterized a heterogeneous population of rare cells and oncosomes in metastatic castrate resistant prostate cancer [28, 29], colorectal cancer [30], breast cancer [31], and urothelial carcinoma [32, 33]. Identification and characterization of a comprehensive profile of rare events may be particularly important in NSCLC given the rarity of CTCs. In this study, longitudinal LBx samples were collected throughout treatment for 10 patients with the primary goal of understanding the cellular and acellular LBx analytes and their dynamics over time for potential utility in clinical monitoring and decision making.

## Materials and methods

### Study design

This study was a single institution study of 10 patients who were diagnosed with NSCLC with metastatic or unresectable disease that was confirmed with pathology. Eligible patients were starting a first or new line of systemic treatment at the time of enrollment. Patients did not have any known severe anemia. The study was conducted according to the guidelines of the Declaration of Helsinki and approved by the Institutional Review Board at the University of Southern California Norris Comprehensive Cancer Center (protocol HS-17-00854 approved on 13 February 2018) and all patients provided written informed consent. Patients were able to leave the study at any time at their own request or were able to withdraw at the discretion of the investigator for safety, behavioral, or administrative reasons. The reasons for discontinuation were documented.

Patient samples were collected from 1/26/2018 to 5/3/2021. Samples were taken prior to initiation of a new or first line therapy, and at follow-up visits coinciding with their treatment schedule to avoid unnecessary blood draws for up to 70 weeks, with a maximum of 7 LBx samples each taken approximately 7–12 weeks apart. Patients were monitored from the time of enrollment to the date of last follow-up and spanned an average of 314.3 (range 27–548) days. Patients were followed for survival analysis in which progression events were confirmed by clinical imaging. A total of 50 normal donor (ND) samples from individuals with no known pathology were used for comparative analysis.

### Blood collection and processing

Peripheral blood samples (average 7 mL) were collected in Cell-Free DNA blood collection tubes (Streck, Omaha, NE) and placed in a temperature stabilization box for transport. All samples were processed by the Convergent Science Institute in Cancer at University of Southern California within 48 h of collection as described previously [43]. In short, blood samples underwent erythrocyte lysis and all the nucleated cells adhered to custom glass slides (Marienfeld, Lauda-Königshofen, Germany) with approximately 3 million cells per slide. Cells were then incubated in 7% BSA, dried, and stored at – 80 °C for subsequent analysis. WBC counts of the samples were determined automatically prior to processing (Medonic M-series hematology Analyzer, Clinical Diagnostic Solutions INC., Fort Lauderdale, FL) allowing for the calculation of cells/mL.

### Immunofluorescent staining

For sample analysis, 2 slides per test were thawed for immunofluorescent staining as previously described [43, 44]. Slides were processed at room temperature using the IntelliPATH FLX™ autostainer (Biocare Medical LLC, Irvine, CA, USA). Briefly, cells were fixed with paraformaldehyde prior to incubation with 2.5 ug/ml of a mouse IgG1 anti-human CD31:Alexa Fluor® 647 mAb (clone: WM59, MCA1738A647, BioRad, Hercules, CA) and 100 ug/ml of a goat anti-mouse IgG monoclonal Fab fragments (115-007-003, Jackson ImmunoResearch, West Grove, PA), permeabilized using 100% cold methanol, followed by an antibody cocktail consisting of mouse IgG1/IgG2a anti-human cytokeratin (CK) 1, 4, 5, 6, 8, 10, 13, 18, and 19 (clones: C-11, PCK-26, CY-90, KS-1A3, M20, A53-B/A2; C2562, Sigma, St. Louis, MO), mouse IgG1 anti-human CK 19 (clone: RCK108, GA61561-2, Dako, Carpinteria, CA), mouse anti-human CD45:Alexa Fluor® 647 (clone: F10-89-4, MCA87A647, AbD Serotec, Raleigh, NC), and rabbit IgG anti-human vimentin (Vim) (clone: D21H3, 9854BC, Cell Signaling, Danvers, MA). Lastly, slides were incubated with Alexa Fluor® 555 goat anti-mouse IgG1 antibody (A21127, Invitrogen, Carlsbad, CA) and 4′,6-diamidino-2-phenylindole (DAPI; D1306, ThermoFisher) prior to mounted with a glycerol-based aqueous mounting media. The HDSCA3.0 workflow includes technical controls throughout the pipeline as previously described [29, 32, 43]. Controls consisted of ND samples spiked with known cell line cells (SK-BR-3 ATCC: HTB-30 and HPAEC ATCC: PCS-100-022) that were processed and analyzed according to standard protocol.

### Detection and classification of rare events

Samples were imaged using automated scanning microscopy at 100 × magnification. Image data sets were analyzed using OCULAR (Outlier Clustering Unsupervised Learning Automated Report) to identify rare event candidates using 761 morphometric parameters [4, 29]. Images of CTC candidates were presented to a hematopathologist-trained technical analyst for manual data reduction and phenotype classification. Rare events were classified into 12 categories (8 cellular and 4 oncosome categories) based on marker expression in the 4 channels. There were 2 types of circulating tumor cell: epi.CTCs and mes.CTCs. Epi.CTCs were classified as containing a nucleus by DAPI morphology, and presenting as CK positive, Vim negative, CD45/CD31 negative. Mes.CTCs were classified as Epi.CTCs with Vim expression. Other rare cells were described using the positive immunofluorescence marker expression in each of the four channels (for example: DAPI|CD45/CD31 = DAPI positive, CD45/CD31 positive, CK negative, Vim negative). Oncosomes were classified as round DAPI negative CK positive events with variable Vim and CD45/CD31 expression, and were observed both free floating and in close proximity to cells (for example: Onc CK|Vim = Oncosome, CK positive, Vim positive, DAPI negative, CD45/CD31 negative).

### Statistical analysis

Cohort level comparisons and longitudinal analysis were performed using python (version 3.8.5) and the Scipy library (version 1.5.0). Statistical comparisons of analyte enumerations at the cohort level were done using the Wilcoxon rank sum test, also known as the Mann–Whitney U test [45, 46]. The Wilcoxon rank sum test was chosen due its non-parametric nature and robustness to outliers. Statistical significance was set at a p-value of 0.05.

PFS was set to the length of time from date enrolled to last follow-up with no documented progression events. OS was set to the length of time from date enrolled to date of death, or end of study date if there was no date of death. Statistical analysis and data visualizations for PFS and OS were created using R software (version 3.6.3) and the survival library (version 3.2-7). Kaplan–Meier curves were used to estimate the survival functions [47]. To compare two survival functions statistically, the log-rank test was used [48–50]. For kinetic PFS analysis, changes in LBx analyte counts were determined using the change between the two blood draws prior to progression, or the last two draws if there was no patient progression. For PFS and OS, we analyzed 16 LBx analytes and groups: total events, total CK expressing cells, total rare cells, total oncosomes, and each individual channel-type classification for cells and oncosomes. For PFS-Kinetics, we analyzed the change in these 16 factors over time. For each of these factors, analyses were performed at each of the three quartiles. Statistical significance was set at a p-value of 0.05. When median survival could not be calculated because the cohort did not reach 50% survival during the study, median survival is reported as N/A.

## Results

### Patient demographics

A total of 10 patients were enrolled into the study at the time of diagnosis prior to first line therapy (n = 7) or at the start of their next line of therapy (n = 3). Patient demographics are described in Table 1. An average of 3.7 draws were collected per patient and analyzed by the HDSCA3.0 workflow (range: 1–7, median: 3). There was an average of 6.64 million white blood cells (WBCs) per mL for patient samples collected (median: 6.25 million, range: 2.5–20.2 million WBCs). An average of 1.23 mL of blood was analyzed per test (median: 1.1 mL, range: 0.34–2.8 mL).

**Table 1:** NSCLC patient demographic and clinical information

Variables	Category	Value n (%)
Age		Range: 30–81 Median: 61
Gender	Female	8 (80%)
	Male	2 (20%)
Race	Caucasian Non-Hispanic	3 (30%)
	Caucasian Hispanic	5 (50%)
	African American	2 (20%)
Cancer history	Yes	1 (10%)
	No	9 (90%)
Family history	Yes	2 (20%)
	No	8 (80%)
Smoking	Yes	3 (30%)
	No	6 (60%)
	N/A	1 (10%)
Disease stage	IIIA	1 (10%)
	IIIB	1 (10%)
	IV*	8 (80%)
Clinical T staging	T1B	3 (30%)
	T2A	1 (10%)
	T3	3 (30%)
	T4	2 (20%)
	N/A	1 (10%)
Clinical N staging	N0	3 (30%)
	N2	2 (20%)
	N3	4 (40%)
	N/A	1 (10%)
Clinical M staging	M0	3 (30%)
	M1A	1 (10%)
	M1B	2 (20%)
	M1C	3 (30%)
	N/A	1 (10%)
Histological subtype	Adenocarcinoma	9 (90%)
	Adenosquamous carcinoma	1 (10%)
Death	Yes	4 (40%)
	N/A	6 (60%)
Prior Therapy	Carboplatin and Taxol	1 (10%)
	Carboplatin and Pemetrexed	1 (10%)
	Pemetrexed	1 (10%)
	None	7 (70%)

*N/A* Data not available

^*^Patient 7 diagnosed as stage IA but metastatic at time of enrollment. Clinical TNM stage reported for diagnosis and not enrollment

### Rare event detection

**Fig. 1:**
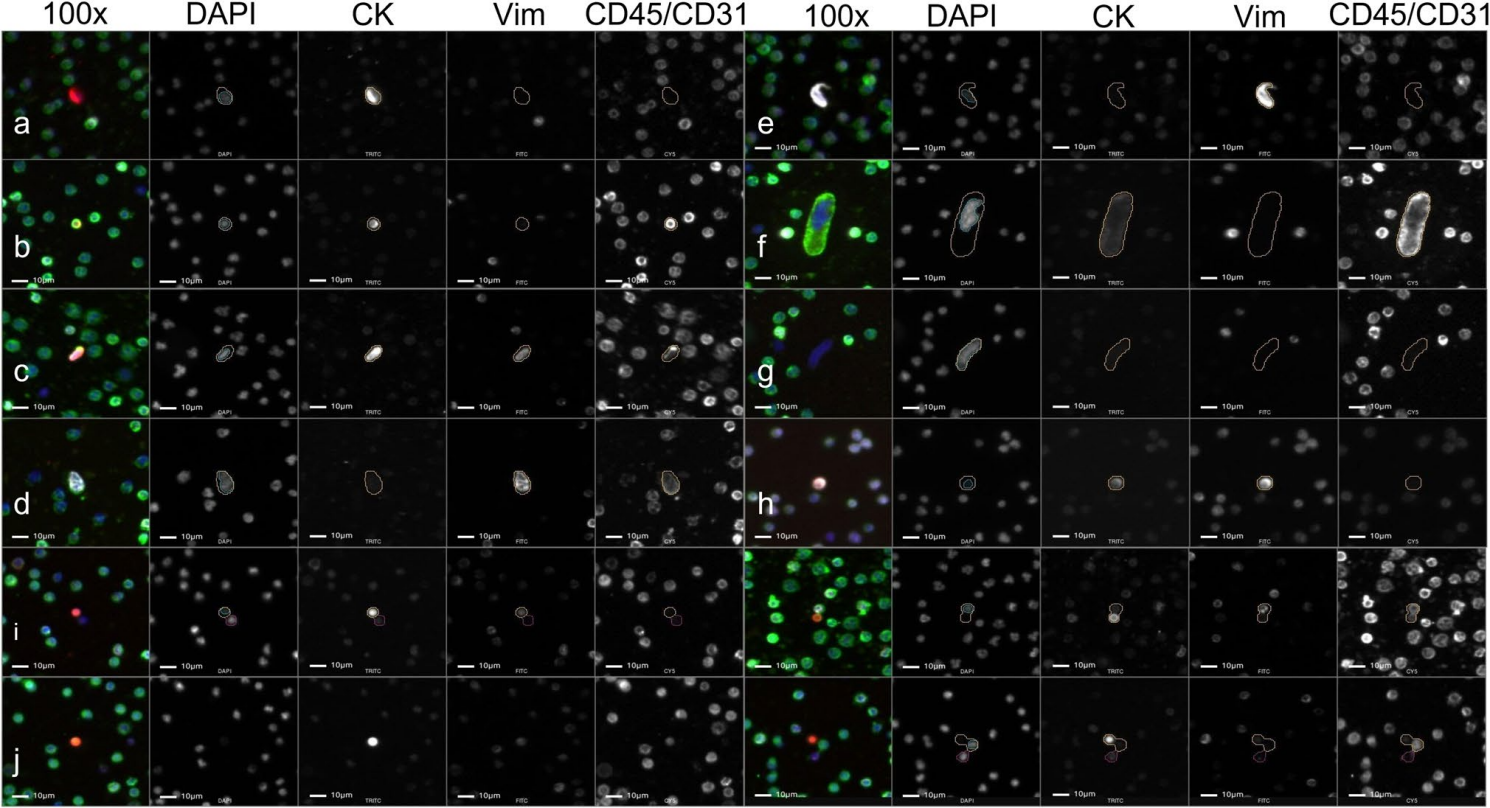
Representative images of rare events detected in the NSCLC patient blood samples. Images taken at 100x magnification. (**a–h**) Rare cells and (**i–l**) oncosomes. DAPI: blue, cytokeratin (**CK**): red, Vim: white, CD45/CD31: green (**a**): Epithelial like CTCs (Epi.CTCs), (**b**): CK|CD45/CD31 cell, (**c**): CK|Vim|CD45/CD31 cell, (**d**): Vim|CD45/CD31 cell, (**e**): Vim only cell, (**f**): CD45/CD31 cell, (**g**): DAPI only cell, (**h**): Mesenchymal like CTCs (Mes.CTCs), (**i**): Onc proximal to cell, (**j**): Onc alone

**Fig. 2:**
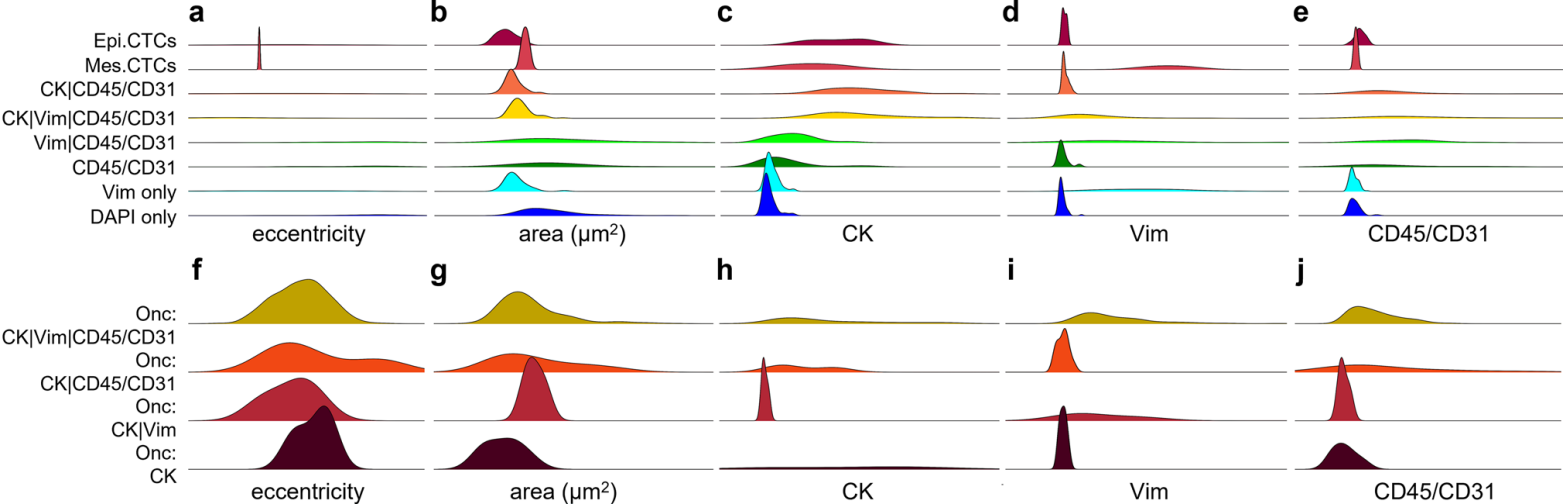
Morphometrics of rare events detected in peripheral blood of NSCLC patients. Probability density distribution plots for morphometric parameters across channel-type classifications for cells (**a–e**) and oncosomes (**f–j**). (**a&f**) eccentricity, (**b&g**) area, (**c&h**) median CK signal intensity, (**d&i**) median Vim signal intensity, (**e&j**) median CD45/CD31 signal intensity

This study analyzed 42 peripheral blood samples collected from 10 patients with stage III-IV NSCLC. A gallery of rare cells and oncosomes are shown in Fig. 1. The identified cellular and oncosome channel-type classifications had considerable morphological and biomarker expression heterogeneity (Fig. 2) suggesting that there may be multiple cellular phenotypes present in each classification group. Oncosomes were detected either alone (*n* = 1077; 35.97%) or in close proximity to cells (*n* = 1917; 64.03%). Additionally, there was a significant correlation between Onc CK|Vim|CD45/CD31 and total oncosomes and a significant correlation between total oncosomes and total cells (Supp. Fig. S1).

### Cohort comparison

**Fig. 3:**
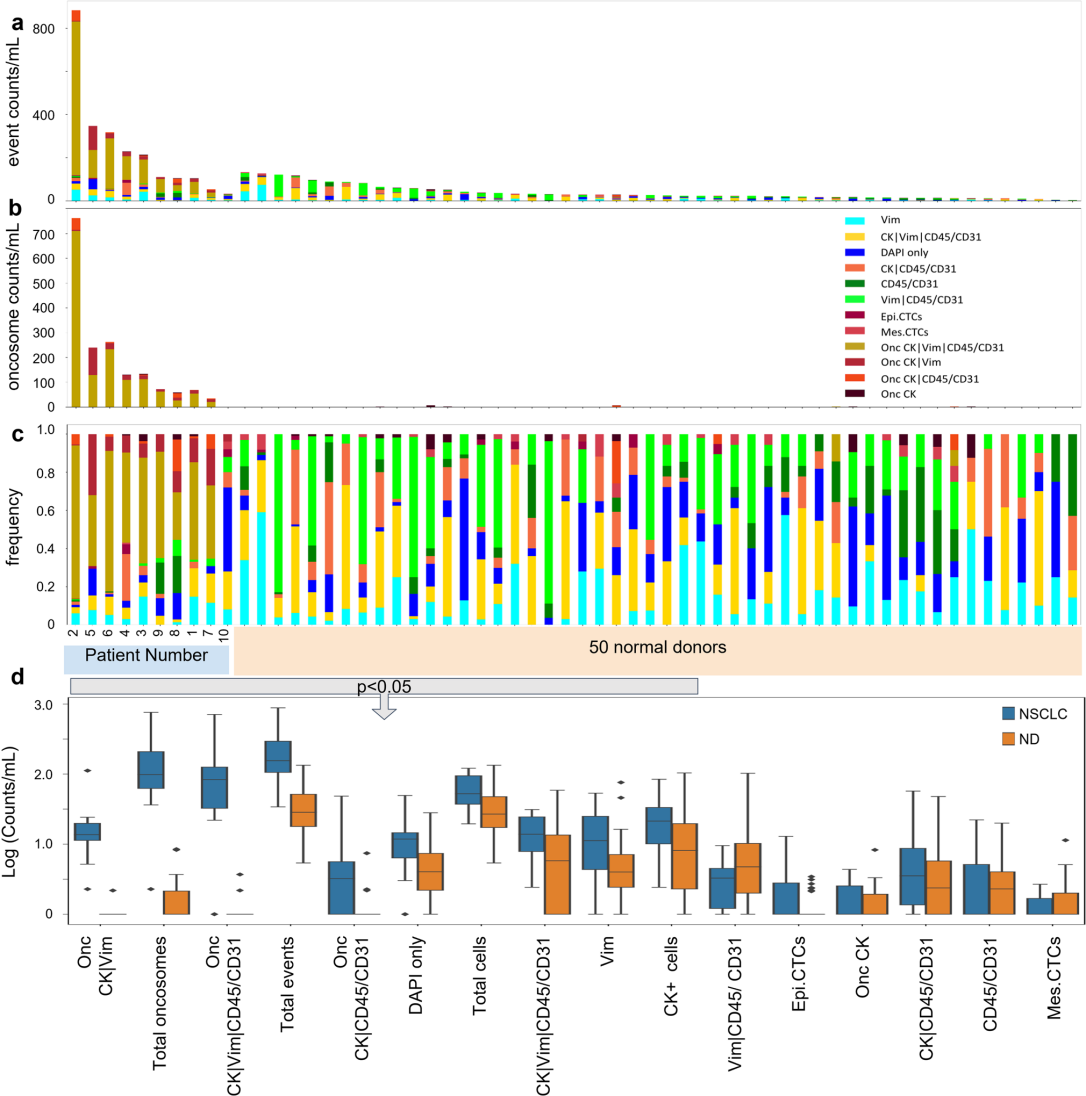
Comparison of rare events detected in the first draws from NSCLC patients and ND by HDSCA3.0. **A** enumeration plot for total events per mL, **B** total oncosome counts per mL, and **C** relative frequency of each rare event classification for patient samples vs ND samples. **D** Box plot depicting differences in logarithmic scale of patients’ first draws as compared to 50 NDs. Factors of significance are marked with red asterisks (*) and outlined by the top bar

To determine if the LBx was distinct in NSCLC patients compared to non-cancerous individuals, the first blood draw collected for each patient was compared to 50 ND draws (Fig. 3). Utilizing the maximum value of specific analytes detected in the ND samples as a threshold for positivity we can quantify and reduce the noise related to the LBx profile. For total events, 5 (50%) NSCLC patients had positive signal in the LBx, 0 (0%) patients had positive signal by total cells, and all (100%) of patients had positive signal using total oncosomes. This suggests that the critical analyte for detecting NSCLC in the LBx is the total oncosome population.

Patient samples had a significantly greater count of total events (p-value < 0.0001), total cells (p-value = 0.0139), total CK expressing cells (p-value = 0.039), as well as specific cellular and acellular channel-type classifications (p-value < 0.05) compared to the ND samples (Fig. 3d). Patient samples had significantly more DAPI-only cells (p-value = 0.0070), CK|Vim|CD45/CD31 cells (p-value = 0.0338), and Vim cells (p-value = 0.0355) as compared to NDs. Similarly, Onc CK|Vim|CD45/CD31 (p-value < 0.0001), Onc CK|Vim (p-value < 0.0001), and Onc CK|CD45/CD31 (p-value = 0.0062) were detected at higher levels in NSCLC patient samples compared to ND. Descriptive statistics for Supplemental Table S1.

### Longitudinal analysis of NSCLC cohort

**Fig. 4:**
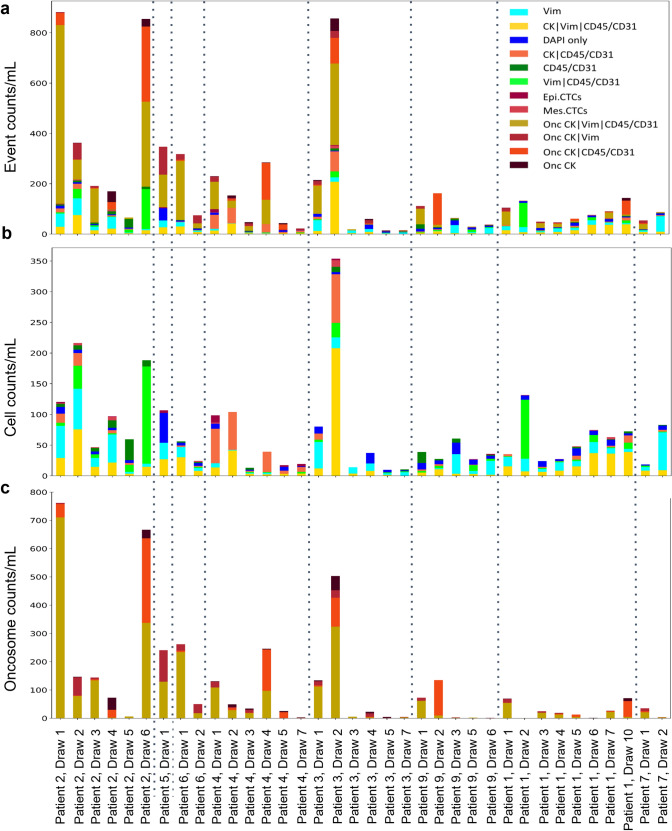
Enumeration frequency plots for all analyzed patient samples presented longitudinally. Patient draws are separated by dashed lines. **A** Total rare event enumerations (events/mL), **B** cellular enumerations (cells/mL) and **C** oncosome enumerations (oncosomes/mL)

As this study included a longitudinal collection of samples per patient, we explored the dynamics of the LBx profile. Enumeration plots for longitudinal patient samples are provided in Fig. 4. Throughout treatment the LBx profile and individual analytes fluctuate potentially as a response to treatment. The 10 patients of this study received 16 different therapeutic agents after their enrollment in this study in which therapeutic compounds were counted individually if they were part of a multi-drug treatment. Therefore, various lines of therapy may affect each patient’s LBx profile. We further analyzed the kinetics of the various analytes in reference to patient outcome (see Survival analysis).

### Survival analysis

To investigate the clinical relevance of the rare events detected in the LBx, we ran PFS and OS analyses of LBx analytes at single time points and longitudinally to assess changes over time. All variables analyzed can be found in Supplemental Table S2. PFS was reported for all patients, in which 4 patients had progression events. At the time of data analysis, 3 patients were confirmed to be deceased (Patient 2, Patient 8, and Patient 9).

PFS was performed using the first draw collected from each patient. Patients with total events, total cells, or total oncosomes above a threshold (162.91, 51.56, 101.85 respectively) at baseline had a longer PFS than those below (p-value = 0.02). Specific phenotypic classification of rare cells and oncosomes were also correlated to PFS: Onc CK|Vim|CD45/CD31, CK|Vim|CD45/CD31 cells, Vim only cells, and CK|CD45/CD31 cells (Supplemental Table S2). Further analysis was used to evaluate the importance of rare event kinetics with patient PFS. The change between the two draws are represented as a positive number if there was an increase, and a negative number if there was a decrease over time. Interestingly, only the change in total oncosome count during therapy was significantly correlated to PFS (p-value = 0.02). Patients with a decrease greater than 31.31 oncosomes/mL over time had significantly shorter PFS than those less than 31.31 oncosomes/mL. Lastly, patients with first draw total oncosome greater than 61.70 events/mL (n = 7) had a longer OS than those who had less than 61.70 events/mL (n = 3; p-value = 0.01).

## Discussion

The LBx has the potential to significantly advance patient care by addressing current clinical challenges in NSCLC as a minimally invasive approach to monitoring disease progression. In the stage III-IV NSCLC cohort presented here, we have identified several LBx analytes unique to the cancer patient as compared to NDs. Survival analysis indicates that a single draw, as well as longitudinal sampling, can inform prognosis. We observed a high inter- and intra-patient heterogeneity suggesting either biological variability in disease or noise due to other conditions. A larger study is necessary to further sample the patient population and evaluate potential signal against disease specific control. Additionally, molecular analysis is required to understand the biological significance of the detected rare events. The data presented here supports the potential clinical utility of the LBx in disease detection and longitudinal monitoring of late-stage NSCLC patients on therapy.

CTC detection rate has varied significantly in NSCLC due to use of different techniques for CTC isolation and enumeration, variable thresholds to discriminate between high- and low-risk patients, and the enrollment of heterogeneous and often small cohorts of patients. Most CTC detection methods use biomarker or size-based enrichment. Recent studies suggest a CTC sensitivity rate of 31.7–78% using CellSearch in late-stage NSCLC [17, 20]. This detection rate is usually lower in early-stage NSCLC, however, there have been limited studies that utilize CTC detection in localized disease settings [8, 34]. The rare cell population detected in this study showed considerable heterogeneity in biomarker expression and morphology. The CTC population (epithelial like CTC [epi.CTC] and mesenchymal like CTC [mes.CTC]) was determined to be minimally detected in our patient cohort and negligible in relation to predicting survival outcomes. This is supported by prior research [21, 35, 36] which found that the presence of EpCAM negative CTCs were found to be associated with shorter survival. Expanding beyond the conventionally defined epithelial positive CTC, and seeking to characterize and understand all subpopulations of rare cells may be critical for understanding the development and progression of disease in NSCLC. Further characterization and understanding of each rare cell population identified in this study is warranted.

This study demonstrates a higher incidence of oncosomes compared to NDs. Further, oncosomes were more prevalent than epi.CTCs and mes.CTCs and determined to be associated with patient PFS and OS. Interestingly, oncosomes were found to correlate with survival both at a single timepoint and over time. Given the rarity of CTCs in NSCLC, the detection of oncosomes through the enrichment-free approach used here is a promising new LBx analyte with potential implications for clinical care. Research has suggested that oncosomes may be capable of spreading tumor promoting material [37], play a role in creating tumor favorable surroundings [38], and aid in the movement of tumor and endothelial cells [39]. Furthermore, a previous paper [28] using similar methodology as presented here found and characterized oncosomes (previously referred to as large extracellular vesicles, LEVs) levels in metastatic castrate resistant prostate cancer. Gerdtsson et al. found that oncosomes were 1.9 times as frequent as CTCs, and that LEVs were identified in 73% of CTC-negative LBx samples. In a more recent paper using the similar methodology as presented here [31], Setayesh et al. analyzed oncosomes in a cohort of breast cancer patients and observed that tracking tumor associated oncosomes allowed for the stratification of early stage breast cancer from NDs, alluding to the clinical utility of these biomarkers. Although the characterization of oncosomes is not yet complete, data suggests these acellular events are important biomarkers for detection and monitoring of disease and further analyses are warranted.

While changes in CTC counts can be associated with patient response [4, 17, 21, 40], it is important to understand CTC dynamics with a clinical timeframe in mind. In a previous publication using a similar methodology as the study here, Shishido et al. found that an increase in CTC counts within the first 3 months of treatment indicated a better PFS as compared to patients with a stable or decreasing CTC profile [4]. This suggests that an increasing CTC profile could suggest response to treatment, while an increase later in the line of therapy may be indicative of tumor growth. Furthermore, factors such as therapy type may also be important for understanding CTC kinetics and their relationship with patient survival. The 10 patients of this study received 16 different therapeutic agents after their enrollment in this study. Each of these therapeutic agents may have a treatment-unique effect on the patient's LBx profiles. Understanding how the various treatments pathways affect disease can be interesting and can shed light on the relationship between rare cell and oncosome groups in clinical response to specific treatment paths.

Circulating tumor DNA (ctDNA) isolated from cfDNA has emerged as another useful analyte, though the sensitivity in detecting NSCLC varies depending on the technology (targeted vs. untargeted) and patient cohort tested. In advanced NSCLC, ctDNA can be used to identify oncogenic driver mutations which may inform treatment decisions using targetable therapy or in real-time monitoring during a patient's treatment course to determine efficacy or mechanisms of resistance. A meta-analysis of several studies with various ctDNA analysis methodologies found an overall sensitivity of 65.7% and specificity of 99.8% for targeted mutation detection [41]. Beyond targeted mutational analyses, structural analysis via copy number alterations and fragmentation of ctDNA can be utilized to detect and monitor NSCLC [42]. Multi-analyte platforms allow for a more comprehensive view of the disease for each patient [13] and combining ctDNA analysis with the cellular analysis conducted in this study may provide new insight into disease progression and biological understanding.

This study demonstrates the utility of an unbiased rare event detection approach to LBx analysis. For the first time, we demonstrate a cancer unique LBx profile that includes multiple types of circulating rare cells and oncosomes from the peripheral blood of NSCLC patients. Given the challenges in detecting CTCs, the presence of oncosomes and their association with patient survival outcomes warrants future larger cohort studies. Further downstream analyses of the LBx analytes by genomics and proteomics would provide additional insight into the biological function of each cellular and acellular event and potential relation to disease state.

### Supplementary Information

**Additional file1** Additional Figure and Table; Figure S1; Table S1.

## Data Availability

All data discussed in this manuscript are included in the main manuscript text or supplementary materials. The imaging data are available through the BloodPAC Data Commons, Accession ID “BPDC000137” (https://data.bloodpac.org/discovery/BPDC000137).

## Code availability

All code developed is standard and easily reproducible.
