# Supplementary material for: Longitudinal tracking of circulating rare events in the liquid biopsy of stage III–IV non-small cell lung cancer patients
Source: Discov Oncol. 2024 May 3;15:142. doi: 10.1007/s12672-024-00984-4 (PMC11068717; doi:10.1007/s12672-024-00984-4)
Supplement: Supplementary file 1 — Additional file1 Additional Figure and Table; Figure S1; Table S1. [file 12672_2024_984_MOESM1_ESM.docx]

**Supplemental Information**

**Patient 1**

JN-01 is a 71-year-old Caucasian female diagnosed with stage IIIA NSCLC Adenocarcinoma (clinical staging: T1b, N2, M0). She had no prior smoking history and was diagnosed via biopsy on 8/5/2016. Patient 1 had a prior therapy of Carboplatin and Taxol until 12/1/2016. A total of 7 blood draws were obtained on 1/26/2018, 4/27/2018, 7/27/2018, 10/3/2018, 12/28/2018, 3/13/2019, and 6/28/2019. From 1/26/2018-3/30/2018, she was placed on a combination therapy of Carboplatin, Pemetrexed, and Pembrolizumab. From 3/6/2019-7/12/2019 she was placed on clinical trial 2N-13-6, consisting of 6 cycles of Azacitidine, Entinostat with Nivolumab as compared to Nivolumab single agent. From 7/31/2019-9/25/2019, she was placed on clinical trial 0C-17-17, which consisted of BMS0986258 (antagonistic antibody, blocks TIM-3 signals in T cells) either (blinded) as a single agent and in combination with Nivolumab. Patient 1 had 4 progressions determined by CT on 12/1/2017, 2/12/2019, 7/10/2019, 9/19/2019. Patient was alive with active cancer on the date of the last follow up on 7/28/2019.

**Patient 2**

Patient 2 is a 70-year-old African American female with a history of prior cancer. Patient did not have a past smoking history. She was diagnosed on 3/4/2016 with stage IV NSCLC Adenocarcinoma (Clinical staging T3, N0, M1a). Six blood draws were obtained on 3/30/2018, 7/6/2018, 8/31/2018, 11/30/2018, 2/22/2019, and 6/12/2019. She received prior therapy of Carboplatin and Pemetrexed until 2/26/2018. She was then placed on 0S-16-8 from 4/6/2018-8/19/2019. She had 2 progressions on 2/7/2018 and 4/26/2019. Patient was last followed up on 7/30/2019, in which she was alive with active cancer. Patient entered hospice and then passed away on 8/19/2019.

**Patient 3**

Patient 3 is a 30-year-old Hispanic female diagnosed with stage IIIB NSCLC Adenocarcinoma (Clinical staging T1b, N3, M0) on 5/29/2018. She had no prior smoking history, personal cancer history or family history. She had no prior therapy and was placed on clinical trial 2N-15-6, from 6/28/2018-10/24/2019. She was then placed on Alectinib from 10/24/2019 till an unknown date. Seven blood draws were collected on 6/21/2018, 9/20/2018, 12/13/2018, 3/7/2019, 5/23/2019, 8/22/2019, and 10/31/2019. At the date of last follow up on 10/31/2019, patient was alive with active cancer.

**Patient 4**

Patient 4 is a 51-year-old Hispanic female who was diagnosed on 4/30/2018 with stage IV NSCLC Adenocarcinoma (Clinical T4, N3, M1c). Patient 4 had a smoking history, but no personal cancer history or family history of cancer. She received no prior therapy and started Erlotinib on 7/1/2018-12/1/2020. She was then placed on Osimertinib from 12/1/2020 until an unknown end date. Six blood draws were collected on 7/19/2018, 10/11/2018, 1/3/2019, 5/2/2019, 6/22/2019, and 12/12/2019. The patient was last followed up on 12/12/2019 when she was alive with active cancer.

**Patient 5**

Patient 5 is a 61-year-old Hispanic female with a family history of cancer. Patient 5 had an unknown smoking history and no prior cancer history. She was diagnosed on 7/3/2018 with stage IV NSCLC Adenocarcinoma (Clinical T3, N0, M1c). Patient had no prior therapy. One blood draw was collected on 8/6/2018. Patient then withdrew from the study and returned to Mexico on 9/12/2018, at which point she was alive with active cancer.

**Patient 6**

Patient 6 is a 56-year-old Caucasian female diagnosed with metastatic stage IV Adenosquamous carcinoma on 2/1/2018 (Clinical T4, N3, M1b). Patient had a smoking history but no previous cancer history and no family cancer history. She was on no prior therapy and started Pembrolizumab on 10/31/2018- 4/2/2019. Two blood draws were collected on 10/31/2018 and 1/23/2019. Patient withdrew from the study and entered hospice on 4/2/2019 at which point she was alive with active cancer.

**Patient 7**

Patient 7 is a 48-year-old Hispanic white female diagnosed with Stage IA (Clinical T2a, N0, M0, pathological T1b, N0) Adenocarcinoma of the lung on 6/26/2017. Patient recurred in 2018 to the liver (Clinical T0, N0, M1 at time of study enrollment). Patient had no prior therapy. She was started on Osimertinib on 1/1/2019 till an unknown end date. Two blood draws were collected on 1/10/2019 and 4/4/2019. Patient withdrew from the study on 4/4/2019 at which point the patient was alive with active disease.

**Patient 8**

Patient 8 is an 81-year-old African American male with a family history of cancer but no smoking history and no personal cancer history. He was diagnosed with stage IV (Clinical T3, N2, M1c) Adenocarcinoma on 1/2/2019. He had no prior therapy and was put on Osimertinib on 1/25/2019-4/10/2019. One blood draw was collected on 1/23/2019. Patient passed away on 4/10/2019.

**Patient 9**

Patient 9 is a 61-year-old Hispanic male diagnosed with stage IV (N/A clinical staging information) Adenocarcinoma on 1/14/2019. Patient had no prior therapy. He started Carboplatin, Pemetrexed, Pembrolizumab from 3/12/2018-5/14/2019. He then started Pemetrexed/Pembrolizumab 6/14/2019-8/6/2019. He then started on Pemetrexed from 8/6/2019-1/21/2020. He had a progression on 12/1/2019. Four blood draws were collected on 2/21/2019, 5/23/2019, 8/1/2019, and 2/27/2020. Patient passed away 5/6/2020.

**Patient 10**

Patient 10 is a 64-year-old Caucasian female with no family history of cancer, smoking history, or personal cancer history. She was diagnosed in 2018 with stage IV (Clinical T1b, N3, M1b) Adenocarcinoma. She was on a prior therapy with Pemetrexed from 7/1/2020-3/10/2021. Patient 10 then started 2N-20-7 (carboplatin-paclitaxel-pevonedistat) from 5/3/2021-8/18/2021, and then received a Vinorelbine from 8/25/2021-10/6/2021. Patient then started Braftovi and Mektovi from 10/18/2021-2/25/2022. Patient had recurrence on 11/29/2021, followed by progression events on 10/7/2021 and 2/15/2022. Four blood draws were obtained on 5/3/2021, 8/25/2021, 11/3/2021, and 2/18/2022. Patient passed away on 3/31/2022.

**
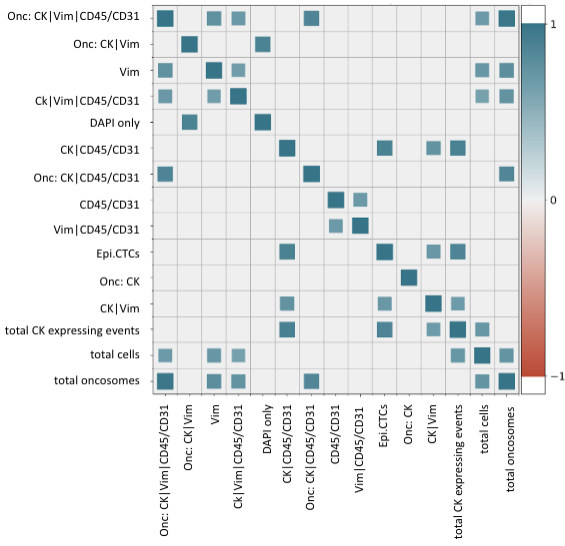
**

**Supplemental Figure 1.** Pearson correlation plot showing statistically significant (p-value < 0.05) rare event correlations for patient samples with red-to-blue color gradient indicating a negative to positive correlation.

**Supplemental Table 1.** Descriptive statistics for each liquid biopsy measurement for the NSCLC and ND cohorts. Top: NSCLC, Bottom: ND.

| Measurement | Average | Median | StDev | Min | Max |
| --- | --- | --- | --- | --- | --- |
| Total events/mL | 240.11 | 162.91 | 249.71 | 33.03 | 882.30 |
|  | 39.70 | 27.55 | 33.88 | 4.39 | 132.94 |
| Total Oncosomes/mL | 176.75 | 101.85 | 222.31 | 1.28 | 762.06 |
|  | 0.76 | 0.00 | 1.56 | 0.00 | 7.48 |
| Total Cells/mL | 63.36 | 51.56 | 35.55 | 18.50 | 120.24 |
|  | 38.94 | 25.91 | 33.89 | 4.39 | 132.94 |
| Total CK/mL | 26.44 | 20.43 | 24.35 | 1.42 | 83.46 |
|  | 15.30 | 7.14 | 20.46 | 0.00 | 102.90 |
| D\|CD | 4.63 | 0.00 | 8.06 | 0.00 | 21.22 |
|  | 2.48 | 1.29 | 3.84 | 0.00 | 18.97 |
| D\|CK | 1.97 | 0.00 | 3.79 | 0.00 | 11.94 |
|  | 0.23 | 0.00 | 0.59 | 0.00 | 2.43 |
| D\|CK\|CD | 9.19 | 2.53 | 17.20 | 0.00 | 56.18 |
|  | 4.75 | 1.37 | 9.40 | 0.00 | 46.86 |
| D\|CK\|V | 0.39 | 0.00 | 0.66 | 0.00 | 1.68 |
|  | 0.75 | 0.00 | 1.70 | 0.00 | 10.38 |
| D\|CK\|V\|CD | 14.88 | 12.89 | 10.43 | 1.42 | 30.18 |
|  | 9.57 | 4.84 | 13.17 | 0.00 | 57.87 |
| D\|V | 17.13 | 11.02 | 18.23 | 0.00 | 52.38 |
|  | 6.28 | 3.01 | 12.02 | 0.00 | 75.11 |
| D\|V\|CD | 2.57 | 2.29 | 2.68 | 0.00 | 8.52 |
|  | 10.39 | 3.74 | 18.43 | 0.00 | 101.71 |
| DAPI-only | 12.59 | 10.81 | 13.57 | 0.00 | 48.51 |
|  | 4.49 | 3.03 | 4.98 | 0.00 | 27.08 |
| Onc CK | 0.90 | 0.00 | 1.31 | 0.00 | 3.37 |
|  | 0.49 | 0.00 | 1.16 | 0.00 | 7.28 |
| Onc CK\|CD | 7.92 | 2.48 | 14.90 | 0.00 | 47.42 |
|  | 0.18 | 0.00 | 0.93 | 0.00 | 6.42 |
| Onc CK\|V | 22.09 | 12.65 | 31.96 | 1.28 | 111.11 |
|  | 0.02 | 0.00 | 0.17 | 0.00 | 1.18 |
| Onc CK\|V\|CD | 145.83 | 85.63 | 209.74 | 0.00 | 710.46 |
|  | 0.08 | 0.00 | 0.41 | 0.00 | 2.69 |

**Supplemental Table 2.** NSCLC patient KM survival outcomes for PFS, OS and PFS Kinetics. Significant factors marked with an asterisk (*). All values are in events/mL.

| Type of Analysis | Factor | Quartile | Median Days of Survival | P-Value |
| --- | --- | --- | --- | --- |
| PFS | > 105.06 events  ≤ 105.06 events | Q1 | NA  296 | 0.2 |
| PFS | > 162.91 events*  ≤ 162.91 events* | Q2 | NA  210 | 0.02* |
| PFS | > 296.11 events  ≤ 296.11 events | Q3 | 392  382 | 1 |
| PFS | > 9.19 CK expressing cells  ≤ 9.19 CK expressing cells | Q1 | 392  84 | 0.08 |
| PFS | > 20.43 CK expressing cells*  ≤ 20.43 CK expressing cells* | Q2 | NA  210 | 0.02* |
| PFS | > 32.31 CK expressing cells  ≤ 32.31 CK expressing cells | Q3 | 392  382 | 0.3 |
| PFS | > 36.26 cells  ≤ 36.26 cells | Q1 | NA  296 | 0.2 |
| PFS | > 51.56 cells*  ≤ 51.56 cells* | Q2 | NA  210 | 0.02* |
| PFS | > 94.03 cells  ≤ 94.03 cells | Q3 | 392  382 | 0.4 |
| PFS | > 61.70 oncosomes  ≤ 61.70 oncosomes | Q1 | 392  210 | 0.4 |
| PFS | > 101.85 oncosomes*  ≤ 101.85 oncosomes* | Q2 | NA  210 | 0.02* |
| PFS | > 213.86 oncosomes  ≤ 213.86 oncosomes | Q3 | 392  382 | 1 |
| PFS | > 33.21 Onc CK\|Vim\|CD45/31  ≤ 33.21 Onc CK\|Vim\|CD45/31 | Q1 | 392  210 | 0.4 |
| PFS | > 85.63 Onc CK\|Vim\|CD45/31*  ≤ 85.63 Onc CK\|Vim\|CD45/31* | Q2 | NA  210 | 0.02* |
| PFS | > 125.04 Onc CK\|Vim\|CD45/CD31  ≤ 125.04 Onc CK\|Vim\|CD45/CD31 | Q3 | 392  382 | 1 |
| PFS | > 0 Onc CK\|CD45/CD31  ≤ 0 Onc CK\|CD45/CD31 | Q1 | 392  210 | 0.5 |
| PFS | > 2.48 Onc CK\|CD45/CD31  ≤ 2.48 Onc CK\|CD45/CD31 | Q2 | 392  296 | 0.3 |
| PFS | > 4.63 Onc CK\|CD45/CD31  ≤ 4.63 Onc CK\|CD45/CD31 | Q3 | 392  382 | 1 |
| PFS | > 6.91 CK\|Vim\|CD45/CD31*  ≤ 6.91 CK\|Vim\|CD45/CD31* | Q1 | 392  147 | 0.009* |
| PFS | > 12.89 CK\|Vim\|CD45/CD31  ≤ 12.89 CK\|Vim\|CD45/CD31 | Q2 | 392  210 | 0.6 |
| PFS | > 24.06 CK\|Vim\|CD45/CD31  ≤ 24.06 CK\|Vim\|CD45/CD31 | Q3 | 392  382 | 1 |
| PFS | > 3.54 Vim*  ≤ 3.54 Vim* | Q1 | 392  147 | 0.009* |
| PFS | > 11.02 Vim  ≤ 11.02 Vim | Q2 | 392  210 | 0.6 |
| PFS | > 24.39 Vim  ≤ 24.39 Vim | Q3 | 392  382 | 0.4 |
| PFS | > 0.29 Vim\|CD45/CD31  ≤ 0.29 Vim\|CD45/CD31 | Q1 | 382  NA | 0.2 |
| PFS | > 2.29 Vim\|CD45/CD31  ≤ 2.29 Vim\|CD45/CD31 | Q2 | 301  382 | 0.5 |
| PFS | > 3.53 Vim\|CD45/CD31  ≤ 3.53 Vim\|CD45/CD31 | Q3 | 392  382 | 0.4 |
| PFS | > 10.34 Onc CK\|Vim  ≤ 10.34 Onc CK\|Vim | Q1 | NA  301 | 0.5 |
| PFS | > 12.65 Onc CK\|Vim  ≤ 12.65 Onc CK\|Vim | Q2 | NA  210 | 0.1 |
| PFS | > 18.97 Onc CK\|Vim  ≤ 18.97 Onc CK\|Vim | Q3 | NA  382 | 0.52 |
| PFS | > 0.60 CK\|CD45/CD31  ≤ 0.60 CK\|CD45/CD31 | Q1 | 392  NA | 0.7 |
| PFS | > 2.53 CK\|CD45/CD31  ≤ 2.53 CK\|CD45/CD31 | Q2 | 392  NA | 0.2 |
| PFS | > 8.26 CK\|CD45/CD31*  ≤ 8.26 CK\|CD45/CD31* | Q3 | NA  210 | 0.03* |
| PFS | > 5.54 DAPI only  ≤ 5.54 DAPI only | Q1 | 210  NA | 0.8 |
| PFS | > 10.81 DAPI only  ≤ 10.81 DAPI only | Q2 | 392  382 | 0.9 |
| PFS | > 13.59 DAPI only  ≤ 13.59 DAPI only | Q3 | 210  392 | 0.2 |
| PFS | > 0 Onc CK  ≤ 0 Onc CK | Q1 & Q2 | 382  210 | 0.1 |
| PFS | > 1.54 Onc CK  ≤ 1.54 Onc CK | Q3 | NA  382 | 0.6 |
| PFS | > 0 CD45/CD31  ≤ 0 CD45/CD31 | Q1 & Q2 | 392  382 | 0.3 |
| PFS | > 4.28 CD45/CD31  ≤ 4.28 CD45/CD31 | Q3 | 238  382 | 0.3 |
| PFS | > 0 mes.CTCs  ≤ 0 mes.CTCs | Q1 & Q2 | 210  382 | 0.9 |
| PFS | > 0.74 mes.CTCs  ≤ 0.74 mes.CTCs | Q3 | 392  382 | 0.9 |
| PFS | > 0 epi.CTCs  ≤ 0 epi.CTCs | Q1 & Q2 | 210  382 | 0.9 |
| PFS | > 1.80 epi.CTCs  ≤ 1.80 epi.CTCs | Q3 | 392  382 | 0.4 |
| PFS-Kinetics | Δ > -98.38 events  Δ ≤ -98.38 events | Q1 | 126  170 | 0.4 |
| PFS-Kinetics | Δ > 11.70 events  Δ ≤ 11.70 events | Q2 | 126  170 | 0.2 |
| PFS-Kinetics | Δ > 14.92 events  Δ ≤ 14.92 events | Q3 | NA  140 | 1 |
| PFS-Kinetics | Δ > -10.14 CK expressing cells  Δ ≤ -10.14 CK expressing cells | Q1 | 126  170 | 0.4 |
| PFS-Kinetics | Δ > 3.69 CK expressing cells  Δ ≤ 3.69 CK expressing cells | Q2 | 132  147 | 0.8 |
| PFS-Kinetics | Δ > 15.08 CK expressing cells  Δ ≤ 15.08 CK expressing cells | Q3 | NA  132 | 0.3 |
| PFS-Kinetics | Δ > -11.85 cells  Δ ≤ -11.85 cells | Q1 | 162  133 | 0.5 |
| PFS-Kinetics | Δ > 8.11 cells  Δ ≤ 8.11 cells | Q2 | 162  133 | 0.6 |
| PFS-Kinetics | Δ > 33.08 cells  Δ ≤ 33.08 cells | Q3 | NA  140 | 1 |
| PFS-Kinetics | Δ > -82.07 oncosomes  Δ ≤ -82.07 oncosomes | Q1 | 126  192 | 0.05 |
| PFS-Kinetics | Δ > -31.31 oncosomes*  Δ ≤ -31.31 oncosomes* | Q2 | 119  192 | 0.02* |
| PFS-Kinetics | Δ > 3.85 oncosomes/mL  Δ ≤ 3.85 oncosomes/mL | Q3 | 119  147 | 0.2 |
| PFS-Kinetics | Δ > -17.97 Onc CK\|Vim\|CD45/CD31  Δ ≤ -17.97 Onc CK\|Vim\|CD45/CD31 | Q1 | 132  NA | 0.3 |
| PFS-Kinetics | Δ > -7.75 Onc CK\|Vim\|CD45/CD31  Δ ≤ -7.75 Onc CK\|Vim\|CD45/CD31 | Q2 | 119  192 | 0.1 |
| PFS-Kinetics | Δ > 4.24 Onc CK\|Vim\|CD45/CD31  Δ ≤ 4.24 Onc CK\|Vim\|CD45/CD31 | Q3 | 119  147 | 0.2 |
| PFS-Kinetics | Δ > -4.83 Onc CK\|CD45/CD31  Δ ≤ -4.83 Onc CK\|CD45/CD31 | Q1 | 126  170 | 0.4 |
| PFS-Kinetics | Δ > 0 Onc CK\|CD45/CD31  Δ ≤ 0 Onc CK\|CD45/CD31 | Q2 | 132  147 | 0.9 |
| PFS-Kinetics | Δ > 5.45 Onc CK\|CD45/CD31  Δ ≤ 5.45 Onc CK\|CD45/CD31 | Q3 | NA  132 | 0.3 |
| PFS-Kinetics | Δ > -7.52 CK\|Vim\|CD45/CD31  Δ ≤ -7.52 CK\|Vim\|CD45/CD31 | Q1 | 126  170 | 0.4 |
| PFS-Kinetics | Δ > 0.64 CK\|Vim\|CD45/CD31  Δ ≤ 0.64 CK\|Vim\|CD45/CD31 | Q2 | 132  147 | 0.8 |
| PFS-Kinetics | Δ > 20.20 CK\|Vim\|CD45/CD31  Δ ≤ 20.20 CK\|Vim\|CD45/CD31 | Q3 | NA  132 | 0.3 |
| PFS-Kinetics | Δ > -11.92 Vim  Δ ≤ -11.92 Vim | Q1 | 132  147 | 1 |
| PFS-Kinetics | Δ > -5.56 Vim  Δ ≤ -5.56 Vim | Q2 | 132  147 | 0.8 |
| PFS-Kinetics | Δ > 2.28 Vim  Δ ≤ 2.28 Vim | Q3 | 192  132 | 0.3 |
| PFS-Kinetics | Δ > -1.00 Vim\|CD45/CD31  Δ ≤ -1.00 Vim\|CD45/CD31 | Q1 | 140  156 | 0.7 |
| PFS-Kinetics | Δ > 0 Vim\|CD45/CD31  Δ ≤ 0 Vim\|CD45/CD31 | Q2 | 140  156 | 0.7 |
| PFS-Kinetics | Δ > 1.62 Vim\|CD45/CD31  Δ ≤ 1.62 Vim\|CD45/CD31 | Q3 | 147  132 | 1 |
| PFS-Kinetics | Δ > -2.04 Onc CK\|Vim  Δ ≤ -2.04 Onc CK\|Vim | Q1 | 133  132 | 0.6 |
| PFS-Kinetics | Δ > 0 Onc CK\|Vim  Δ ≤ 0 Onc CK\|Vim | Q2 | 192  132 | 0.3 |
| PFS-Kinetics | Δ > 1.67 Onc CK\|Vim  Δ ≤ 1.67 Onc CK\|Vim | Q3 | NA  140 | 1 |
| PFS-Kinetics | Δ > -1.71 CK\|CD45/CD31  Δ ≤ -1.71 CK\|CD45/CD31 | Q1 | 132  147 | 0.9 |
| PFS-Kinetics | Δ > 2.41 CK\|CD45/CD31  Δ ≤ 2.41 CK\|CD45/CD31 | Q2 | 132  133 | 0.6 |
| PFS-Kinetics | Δ > 5.36 CK\|CD45/CD31  Δ ≤ 5.36 CK\|CD45/CD31 | Q3 | 132  133 | 0.6 |
| PFS-Kinetics | Δ > -8.15 DAPI only  Δ ≤ -8.15 DAPI only | Q1 | 140  104 | 0.9 |
| PFS-Kinetics | Δ > 0.95 DAPI only  Δ ≤ 0.95 DAPI only | Q2 | 132  147 | 0.9 |
| PFS-Kinetics | Δ > 4.95 DAPI only  Δ ≤ 4.95 DAPI only | Q3 | 162  133 | 0.6 |
| PFS-Kinetics | Δ > 0 Onc CK  Δ ≤ 0 Onc CK | Q1 & Q2 | 119  147 | 0.6 |
| PFS-Kinetics | Δ > 3.17 Onc CK  Δ ≤ 3.17 Onc CK | Q3 | NA  132 | 0.3 |
| PFS-Kinetics | Δ > 0.82 CD45/CD31  Δ ≤ 0.82 CD45/CD31 | Q1 | 140  119 | 0.7 |
| PFS-Kinetics | Δ > 1.70 CD45/CD31  Δ ≤ 1.70 CD45/CD31 | Q2 | 147  119 | 0.6 |
| PFS-Kinetics | Δ > 4.17 CD45/CD31  Δ ≤ 4.17 CD45/CD31 | Q3 | 147  132 | 1 |
| PFS-Kinetics | Δ > -1.28 mes.CTCs  Δ ≤ -1.28 mes.CTCs | Q1 | 132  147 | 0.9 |
| PFS-Kinetics | Δ > 0 mes.CTCs  Δ ≤ 0 mes.CTCs | Q2 | 126  170 | 0.2 |
| PFS-Kinetics | Δ > 1.62 mes.CTCs  Δ ≤ 1.62 mes.CTCs | Q3 | 119  147 | 0.2 |
| PFS-Kinetics | Δ > -0.95 epi.CTCs  Δ ≤ -0.95 epi.CTCs | Q1 | 132  147 | 0.9 |
| PFS-Kinetics | Δ > 0 epi.CTCs  Δ ≤ 0 epi.CTCs | Q2 & Q3 | NA  140 | 1 |
| OS | > 105.06 events  ≤ 105.06 events | Q1 | 507  218 | 0.8 |
| OS | > 162.91 events  ≤ 162.91 events | Q2 | 507  440 | 0.2 |
| OS | > 296.11 events  ≤ 296.11 events | Q3 | 507  NA | 0.9 |
| OS | > 9.19 CK expressing cells  ≤ 9.19 CK expressing cells | Q1 | NA  440 | 0.07 |
| OS | > 20.43 CK expressing cells  ≤ 20.43 CK expressing cells | Q2 | 507  440 | 0.2 |
| OS | > 32.31 CK expressing cells  ≤ 32.31 CK expressing cells | Q3 | 507  440 | 0.4 |
| OS | > 36.26 cells  ≤ 36.26 cells | Q1 | 507  218 | 0.8 |
| OS | > 51.56 cells  ≤ 51.56 cells | Q2 | 507  440 | 0.2 |
| OS | > 94.03 cells  ≤ 94.03 cells | Q3 | 507  440 | 0.5 |
| OS | > 61.70 oncosomes*  ≤ 61.70 oncosomes* | Q1 | NA  218 | 0.01* |
| OS | > 101.85 oncosomes  ≤ 101.85 oncosomes | Q2 | 507  440 | 0.2 |
| OS | > 213.86 oncosomes  ≤ 213.86 oncosomes | Q3 | 507  NA | 0.9 |
| OS | > 33.21 Onc CK\|Vim\|CD45/CD31  ≤ 33.21 Onc CK\|Vim\|CD45/CD31 | Q1 | NA  218 | 0.1 |
| OS | > 85.63 Onc CK\|Vim\|CD45/CD31  ≤ 85.63 Onc CK\|Vim\|CD45/CD31 | Q2 | 507  440 | 0.2 |
| OS | > 125.04 Onc CK\|Vim\|CD45/CD31  ≤ 125.04 Onc CK\|Vim\|CD45/CD31 | Q3 | 507  NA | 0.9 |
| OS | > 0 Onc CK\|CD45/CD31  ≤ 0 Onc CK\|CD45/CD31 | Q1 | 507  218 | 0.7 |
| OS | > 2.48 Onc CK\|CD45/CD31  ≤ 2.48 Onc CK\|CD45/CD31 | Q2 | 507  440 | 0.7 |
| OS | > 4.63 Onc CK\|CD45/CD31  ≤ 4.63 Onc CK\|CD45/CD31 | Q3 | 507  NA | 0.3 |
| OS | > 6.91 CK\|Vim\|CD45/CD31*  ≤ 6.91 CK\|Vim\|CD45/CD31* | Q1 | NA  218 | 0.006* |
| OS | > 12.89 CK\|Vim\|CD45/CD31  ≤ 12.89 CK\|Vim\|CD45/CD31 | Q2 | NA  440 | 0.07 |
| OS | > 24.06 CK\|Vim\|CD45/CD31  ≤ 24.06 CK\|Vim\|CD45/CD31 | Q3 | 507  NA | 0.9 |
| OS | > 3.54 Vim*  ≤ 3.54 Vim* | Q1 | NA  218 | 0.006* |
| OS | > 11.02 Vim  ≤ 11.02 Vim | Q2 | 507  440 | 0.2 |
| OS | > 24.39 Vim  ≤ 24.39 Vim | Q3 | 507  440 | 0.8 |
| OS | > 0.29 Vim\|CD45/CD31  ≤ 0.29 Vim\|CD45/CD31 | Q1 | 507  NA | 0.3 |
| OS | > 2.29 Vim\|CD45/CD31  ≤ 2.29 Vim\|CD45/CD31 | Q2 | 440  NA | 0.06 |
| OS | > 3.53 Vim\|CD45/CD31  ≤ 3.53 Vim\|CD45/CD31 | Q3 | 507  440 | 0.5 |
| OS | > 10.33 Onc CK\|Vim  ≤ 10.33 Onc CK\|Vim | Q1 | NA  362 | 0.4 |
| OS | > 12.65 Onc CK\|Vim*  ≤ 12.65 Onc CK\|Vim* | Q2 | NA  440 | 0.02* |
| OS | > 18.97 Onc CK\|Vim  ≤ 18.97 Onc CK\|Vim | Q3 | NA  507 | 0.3 |
| OS | > 0.60 CK\|Vim\|CD45/CD31  ≤ 0.60 CK\|Vim\|CD45/CD31 | Q1 | 507  77 | 0.06 |
| OS | > 2.53 CK\|Vim\|CD45/CD31  ≤ 2.53 CK\|Vim\|CD45/CD31 | Q2 | NA  440 | 0.1 |
| OS | > 8.26 CK\|Vim\|CD45/CD31  ≤ 8.26 CK\|Vim\|CD45/CD31 | Q3 | 507  440 | 0.3 |
| OS | > 5.54 DAPI only  ≤ 5.54 DAPI only | Q1 | 474  NA | 0.2 |
| OS | > 10.81 DAPI only  ≤ 10.81 DAPI only | Q2 | 362  NA | 0.2 |
| OS | > 13.59 DAPI only*  ≤ 13.59 DAPI only* | Q3 | 148  NA | 0.004* |
| OS | > 0 Onc CK  ≤ 0 Onc CK | Q1 & Q2 | NA  440 | 0.2 |
| OS | > 1.54 Onc CK  ≤ 1.54 Onc CK | Q3 | NA  474 | 0.7 |
| OS | > 0 CD45/CD31  ≤ 0 CD45/CD31 | Q1 & Q2 | 440  NA | 0.1 |
| OS | > 4.28 CD45/CD31  ≤ 4.28 CD45/CD31 | Q3 | 440  NA | 0.09 |
| OS | > 0 mes.CTCs  ≤ 0 mes.CTCs | Q1 & Q2 | 507  NA | 0.9 |
| OS | > 0.74 mes.CTCs  ≤ 0.74 mes.CTCs | Q3 | 507  NA | 0.9 |
| OS | > 0 epi.CTCs  ≤ 0 epi.CTCs | Q1 & Q2 | 507  NA | 0.9 |
| OS | > 1.80 epi.CTCs  ≤ 1.80 epi.CTCs | Q3 | 507  440 | 0.5 |
